# Combined Maternal and Post-Hatch Dietary Supplementation of 25-Hydroxycholecalciferol Alters Early Post-Hatch Broiler Chicken Duodenal Macrophage and Crypt Cell Populations and Their Mitotic Activity

**DOI:** 10.3389/fvets.2022.882566

**Published:** 2022-04-11

**Authors:** Samuel F. Leiva, Luis P. Avila, Gerardo A. Abascal-Ponciano, Joshua J. Flees, Kelly M. Sweeney, Jeanna L. Wilson, Jessica D. Starkey, Charles W. Starkey

**Affiliations:** ^1^Department of Poultry Science, Auburn University, Auburn, AL, United States; ^2^Department of Poultry Science, The University of Georgia, Athens, GA, United States

**Keywords:** vitamin D, 25-hydroxycholecalciferol, intestinal innate immunity, macrophage, broiler chicken, pro-inflammatory macrophages, intestinal crypt cell mitotic activity

## Abstract

The previous work has demonstrated that maternal supplementation of the circulating metabolite of vitamin D3 (D3), 25-hydroxycholecalciferol (25OHD3), enhances the immunocompetence of broiler chick offspring. In post-hatch broiler diets, 25OHD3 has been shown to affect intestinal morphology and improve the immune status of broilers. An experiment with a 2 × 2 factorial treatment arrangement was conducted to assess the effects of combining maternal (MDIET) and post-hatch (PDIET) dietary 25OHD3 inclusion on duodenal crypt and macrophage cell populations and mitotic activity in young broiler chickens. All diets were formulated to provide 5,000 IU of vitamin D. Broiler breeder hens were offered 1 of 2 MDIET: 5,000 IU D3 per kg of feed (MCTL) or 2,240 IU of D3 + 2,760 IU of 25OHD3 per kg of feed (M25OHD3) from week 25 to 41. Male broiler offspring (*n* = 480) hatched from eggs collected during week 41 of breeding age were allotted in raised floor pens (4 birds per pen from day 0 to 7 and individually allotted from day 8 to 21). Chicks were fed 1 of 2 PDIET (starter day 0 to 21): 5,000 IU D3 per kg of feed (PCTL) or 2,240 IU D3 + 2,760 IU 25OHD3 (P25OHD3). DUO samples (*n* = 12 birds per treatment per day) were collected on days 3, 6, 9, 12, 15, 18, and 21 for cryohistological and immunofluorescence analysis to facilitate the enumeration of the total macrophages, CD80+ macrophages (pro-inflammatory macrophages), and mitotically active cells (BrdU+) to calculate the proportion of proliferating cells (PPC) per duodenal crypt. Bird age impacted crypt PPC with the greatest PPC per duodenal crypt observed on days 3 and 9, and the lowest PPC per crypt was observed on day 21 (*P* < 0.0001). Broilers from the M25OHD3:PCTL treatment had a greater PPC (*P* =.002) than birds from the MCTL:PCTL treatment at day 3. An interaction among MDIET and PDIET was observed for proliferating macrophages at day 21 (*P* = 0.029) where M25OHD3:P25OHD3 birds had more proliferating macrophages than M25OHD3:PCTL-fed birds. These results indicate that combined MDIET and PDIET 25OHD3 supplementation may alter early post-hatch duodenal development and innate immunity.

## Introduction

Vitamin D is commonly supplied in poultry diets as cholecalciferol (D3). In the chick, most of the absorption of D3 is achieved in the upper jejunum (1). In the bloodstream, Vitamin D binds to the Vitamin D Binding Protein (DBP) for transport during metabolism and to target organs (2). Vitamin D3 does not have any known biological activity, it must be activated through a two-step hydroxylation process (3, 4). D3 is stored in the liver where it is hydroxylated in the 25th position by 25-hydroxylase to produce 25-hydroxycholecalciferol (25OHD3) (5). This hydroxylation process of vitamin D is weakly regulated and levels of 25OHD3 increase proportionally to vitamin D intake (6). For this reason, 25OHD3 is the major circulating form of vitamin D in the bloodstream (7) and is recognized as one of the best indicators of vitamin D status in animals and humans (3, 5).

Previous research reports that supplementation of broiler diets with 25OHD3 can improve productive parameters. Bar et al. reported that 25OHD3 is more efficiently absorbed than D3 in the duodenum of broiler chickens (1). Other studies found that replacement of D3 for 25OHD3 improved average body weight, decreased adjusted feed efficiency, and did not affect bird mortality (8). Dose response studies revealed maximal weight gain, feed efficiency, and breast meat yield achieved with 25OHD3 dietary inclusion ranging from 50 to 70 μg per kg of feed (8). Further, the dietary inclusion of 2,760 IU (69 μg per kg) in diets containing D3 improves cellular and humoral immune responses in addition to bone calcium deposition in 21-day-old broilers (9). The inclusion of 25OHD3 in broiler diets affects the intestinal morphology of growing broilers (10) and the important effects of 25OHD3 have been observed in the immune proficiency of diverse poultry species under immunological challenges (11–13).

Positive effects of 25OHD3 supplementation of maternal diets on the immune status and intestinal morphologic development of chicks have also been reported (14, 15). Saunders-Blades and Korver observed that supplementing broiler breeder diets with 2,760 IU per kg (69 μg) of 25OHD3 improved the vitamin D3 status of chicks upon hatching and increased the efficiency of leukocytes in battling bacterial infection in addition to having greater oxidative burst activity (15). Evidence suggests that the inclusion of 25OHD3 in either broiler or breeder diets may affect the immune function and intestinal morphology of chicks; however no previous studies have evaluated the effect of the combination of maternal and post-hatch supplementation on these parameters. Therefore, in this study we aimed to evaluate the effect of combining maternal and post-hatch dietary 25OHD3 on duodenal crypt cell proliferation and local intestinal innate immunity of young broiler chickens.

## Materials and Methods

The Auburn University Institutional Animal Care and Use Committee approved the use of live birds in this experimental protocol (PRN 2017-3212).

### Breeder Hen Management

Ross 708 broiler breeder pullets (*n* = 350, Aviagen Group, Huntsville, AL) were reared up to 21 weeks of age in a rearing facility and fed starter, developer, and pre-breeder diets without 25OHD3 metabolite in a skip a d restricted feeding program. Pullets were exposed to a photoperiod of 8 h of light per day. They received an *in ovo* vaccination for Marek's disease and were vaccinated on day 1 for reovirus and *Eimeria*. Newcastle, bronchitis, reovirus, and chicken infectious anemia vaccines were applied during the rearing period according to commercial recommendations. The temperature was set at 29°C at placement and gradually reduced to 21°C by the end of the rearing period. At 21 weeks of age, broiler breeder hens were allotted in groups of 35 hens per pen (*n* = 10 pens) along with 3 broiler breeder Yield Plus roosters (Aviagen Group) at a density of 0.22 m^2^ per bird. Breeders were kept at a temperature of 20°C and photo stimulated with 15.25 h of light per day. After 25 weeks of age, broiler breeder hens and roosters were fed 1 of 2 corn and soybean-meal based maternal treatment diets (MDIET; Supplementary Table 1) 5,000 IU of D3 per kg of feed (MCTL; *n* = 5 pens), or 2,240 IU of D3 (Rovimix D3^®^ 500; DSM Nutritional Products Inc., NJ, USA) + 2,760 IU of 25OHD3 (69 μg Rovimix Hy-D^®^ DSM Nutritional Products Inc.) per kg of feed (M25OHD3; *n* = 5 pens). Hen feeding was restricted to between 110 and 170 g per bird per day based on egg production. Clean water was offered *ad libitum*.

### Broiler Chicken Hatching and Management

Hatching eggs from 41-week-old broiler breeder hens from both MDIET treatments were collected and set for incubation at 37.5°C and a relative humidity of 53%; the eggs were rotated 45°C every h and hatched at a temperature of 37°C. On the day of hatch, unvaccinated male broiler chicks (*n* = 480) from the 2 maternal treatments were identified with a wing tag, individually weighed to determine day 0 body weight and randomly allotted to raised floor pens (*n* = 120) at a stocking density of 0.05 m^2^ per bird (*n* = 4 birds per pen). On day 7, broilers were redistributed to individual pens (*n* = 360 pens; *n* = 90 pens per MDIET × PDIET treatment) with their corresponding dietary treatment (*n* = 1 bird per pen; 0.21 m^2^ per bird). Each pen was equipped with 2 nipple drinkers and 1 galvanized metal feeder. A supplemental plastic feeder was included in the pen from day 1 to 7 to improve feed consumption. Ambient temperature was set to 32°C at placement and gradually reduced to 21°C by day 21 based on bird comfort. From day 0 to 7, birds were reared with a photoperiod of 23 h of light and 1 h of dark with a light intensity of 30 lux; from day 8 to 21, lighting schedule was adjusted to 23 h of light and 1 h of dark with a light intensity of 10 lux.

Broilers were fed one of two broiler post-hatch corn and soybean-meal based treatment diets (PDIET; Supplementary Table 2): 5,000 IU of D3 per kg of feed (PCTL), or 2,240 IU of D3 (Rovimix D3 500; DSM Nutritional Products Inc., NJ, USA) + 2,760 IU of 25OHD3 (69 μg Rovimix Hy-D; DSM Nutritional Products Inc.) per kg of feed (P25OHD3). Feed was provided as a starter crumble diet throughout the experiment (day 1–21) and both feed and water were offered *ad libitum*. Combining of these 2 main effects (MDIET × PDIET) resulted in 4 treatments: MCTL:PCTL, MCTL:P25OHD3, M25OHD3:PCTL, and M25OHD3:P25OHD3. Therefore, this experiment had a randomized complete block design with a 2 × 2 factorial arrangement. Pens were blocked by location. Each block was comprised by 4 pens; each pen represented one treatment. All maternal and post-hatch diets were analyzed by the DSM Nutritional Products TMAS laboratory (Beldivere, NJ). The vitamin D content of diets was measured with AOAC official method 2011.12. The concentration of 25OHD3 in the feed was measured using high performance liquid chromatography (HPLC) and mass spectroscopy procedure.

### Broiler Chicken Growth Performance

Bird mortality and comfort were monitored twice daily. Birds were individually weighed (BW) and pen feeder weights were recorded on day 0, day 7, and on each sampling day to calculate individual mortality corrected bodyweight gain (MCBWG), mortality corrected average daily bodyweight gain (MCADBWG), mortality corrected feed intake (MCFI), and mortality corrected feed conversion ratio (MCFCR).

### Egg Yolk and Blood Plasma 25-Hydroxycholecalciferol Concentration Analysis

Eggs laid by 41-week-old breeder hens from both maternal treatments were collected (*n* = 10 per MDIET; *n* = 2 per pen), yolks were separated and stored at −80°C for further analysis. On the same day of egg collection, blood samples were collected from the brachial vein of breeder hens (*n* = 5 per MDIET treatment). Blood samples from male broiler chicks were collected using cardiac puncture procedure at all ages. Blood samples from chicks of both MDIET treatments were collected on day 0 and from the four MDIET × PDIET treatments on days 3, 6, 9, 12, 15, 18, and 21. Blood samples were collected using a 4.5 ml S-Monovette syringe with lithium heparin beads (Sarstedt, Inc., NC) and 50 mm × 21 mm gauge needle (days 0, 3, 6, 9, and 12) or a 40 mm × 16 mm gauge needle (breeder hens and broilers from day 15 to 21). All blood samples were stored in the same syringes and kept in ice. No more than 6 h after collection, all blood samples were centrifuged at 1,000 RFC at 4°C for 10 min, and plasma was stored at −80°C to await analysis. Plasma and yolk samples were analyzed by DSM Nutritional Products TMAS laboratory (Beldivere, NJ) to determine 25OHD3 concentrations using an isotope dilution assay and HPLC quantification procedure.

### Bromodeoxyuridine Injection and Tissue Sample Collection

On each sampling day, a total of 48 birds (*n* = 12 per MDIET × PDIET treatment from 12 blocks) were randomly selected and intraperitoneally injected with a solution of 5'-bromo-2'-deoxyuridine (BrdU; Alfa Aesar, Haverhill, MA; pH of 8.0; 100 μg of BrdU per g of body weight) at a concentration of 25 mg per ml and placed in disposable containers for 1 h to allow for incorporation of BrdU into mitotically active cells (16, 17). After 1 h, blood was collected from each bird *via* cardiac puncture and birds were immediately euthanized by cervical dislocation. After euthanasia, tissue samples were collected from the duodenum just distal to the duodenal loop to prevent inclusion of pancreatic tissue. Duodenal samples were flushed with tris buffered saline (TBS; pH 7.4; Thermo Fisher Scientific, Fair Lawn, NJ) to remove intestinal contents. Samples were cut lengthwise, coated in magnesium silicate, flash frozen in liquid nitrogen and stored at −80°C until further analysis.

### Cryohistology

Samples were stored at −20°C for 24 h prior to cryohistological analysis. Duodenum samples were embedded in a frozen section compound (VWR International, Westchester, PA) and 5 μm-thick cryosections were collected transverse to the length of intestine using a CM 1950 cryomicrotome (Leica, Nussloch, Germany). Cryosections were then mounted on positively charged slides (VWR International; 5 cryosections per slide) and stored at 4°C for no more than 24 h before immunofluorescence staining.

### Immunofluorescence Staining

All staining procedures were conducted at room temperature. Slides were rehydrated in 5% 20X Tris-buffer saline in an ultra-pure water solution (TBS; Thermo Fisher Scientific) for 10 min. Next, slides were fixed for 10 min in 4% paraformaldehyde in PBS pH 7.4 (Santa Cruz Biotechnology, Santa Cruz, CA) followed by two rinses in TBS. Remaining staining procedures were conducted in a humidified black box. To permeabilize tissue, slides were incubated for 10 min in 0.5% TritonX-100 (Thermo Fisher Scientific). Slides were incubated in 1N hydrochloric acid (VWR International) for 10 min to denature DNA and facilitate BrdU detection followed by a single rinse in TBS. Slides were incubated for 30 min in a blocking solution consisting of 10% horse serum (Sigma-Aldrich, St. Louis, MO) and 0.5% bovine serum albumen (VWR International) in a 0.2% TritonX-100 solution. Primary antibodies were diluted in a blocking solution and slides were incubated in the solution for 1 h followed by three 5 min rinses in TBS. In a similar manner, secondary antibodies were diluted in a blocking solution and slides were incubated for 30 min in the solution followed by three 5 min rinses in TBS. Cryosections were then treated with a 4',6-diamidino-2-phenylindole solution (DAPI; 100 μg per ml; EMD Biosciences, Inc., San Diego, CA) diluted 1:1,000 in TBS and immediately rinsed twice in TBS. Slides were mounted using a Fluoro-gel media containing Tris-buffer (Electron Microscopy Sciences, Hatfield, PA), and glass coverslips (Thermo Fisher Scientific). Finally, slides were permanently sealed with clear fingernail polish (Sally Hansen, New York, NY) and left to dry at room temperature. All cryosections were digitally imaged within 24 h of immunofluorescence staining.

### Primary and Secondary Antibodies

The primary antibodies used were as follows: mouse monoclonal IgG1 anti-chicken monocyte macrophage (KUL01; 1:500 dilution; Cat. No. SC52603; Santa Cruz Biotechnology; Santa Cruz, CA) as a general macrophage marker (18). Armenian hamster monoclonal IgG anti-CD80 (16-10A1 Cat. No. 14-0801-85; Invitrogen, Thermo Fisher Scientific Inc, Waltham, MA), and mouse monoclonal IgG2a anti-BrdU (dilution 1:750 for day 3, 1:3,000 for days 6 and 9; 1:2,500 for day 12; 1:2,000 for day 15; 1:1,000 for day 18, and 1:500 for day 21; Cat. No. MA3-071; Invitrogen, Thermo Fisher Scientific Inc., Waltham, MA). Primary antibodies were detected using the following secondary antibodies (dilution 1:1,000; Invitrogen): Alexa-Fluor 488–conjugated goat anti-mouse IgG1 (Cat. No. A21121), Alexa-Fluor 546–conjugated goat anti-hamster IgG (Cat. No. A2111-0), and Alexa-Fluor 633–conjugated goat anti-mouse IgG2a (Cat. No. A21136).

### Fluorescence Microscopy and Image Analysis

**Figure 1:**
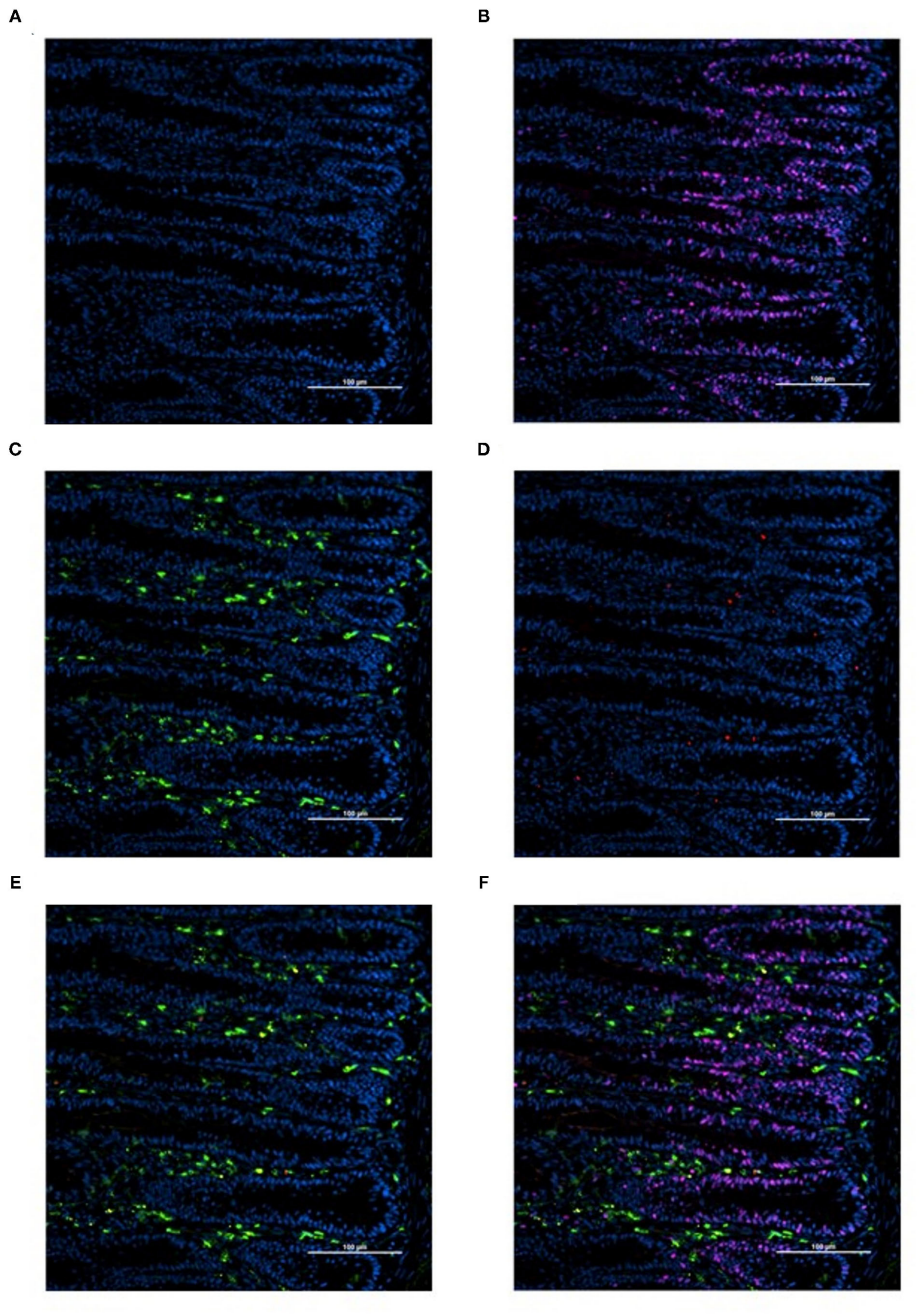
Representative images illustrating the determination of total, proinflammatory, and proliferating macrophage density by immunofluorescence in duodenal cryosections from 18-days-old post-hatch broiler chickens **(A-F)**. **(A)** cell nuclei (blue, DAPI+). **(B)** Proliferating cells (pink, BrdU+; blue, DAPI+). **(C)** Total macrophages, includes pro and anti-inflammatory macrophages (green, KUL01+; blue, DAPI+). **(D)** Proinflammatory macrophages (red, CD80+; blue, DAPI+). **(E)** Proinflammatory and total macrophage merge (red, CD80+; green, KUL01+; blue, DAPI+). **(F)** Merge (red, CD80+; green, KUL01+; pink, BrdU+; blue, DAPI+). Scale bar = 100 μm.

Slides were imaged at 200-fold magnification with an Eclipse Ti-U inverted fluorescence microscope (Nikon Instruments Inc., Melville, NY) with a solid-state LED light-source (Lumencor SOLA Sm II 365 light engine). Two representative images per slide were captured with an incorporated Evolve 512 EMCCF digital camera (Teledyne, Photometrics, Tucson, AZ). One image per slide (*n* = 1 per bird) was analyzed using NIS-Elements Advanced Research Software (Nikon Instruments Inc.) focusing on the crypt region of the tissue section (Figure 1). Cell populations with BrdU+ (proliferating cells; Figure 1B), KUL01+ (total macrophages; Figure 1C), KUL01+;CD80+ (proinflammatory macrophages; Figure 1E), KUL01+;BrdU+ (proliferating macrophages), and KUL01+;BrdU+:CD80+ (proliferating proinflammatory macrophages; Figure 1F) were enumerated. To determine the proportion of proliferating cells per crypt, two well defined crypts were selected from each image (*n* = 2 per bird); the crypt area was defined by drawing a polygon around the epithelium. All cell nuclei and BrdU+ cells within the designated crypt were enumerated and the proportion of proliferating cells per crypt was then calculated by dividing the number of proliferating cells by the total number of cells in the crypt.

### Statistical Analysis

The data were analyzed as a 2-way ANOVA using the generalized linear mixed model (GLIMMIX) procedure of the Statistical Analysis Software (SAS; V9.4; SAS Institute Inc., NC, USA) in which MDIET, PDIET, and MDIET × PDIET were the fixed effects. The Satterthwaite adjustment was used to correct degrees of freedom and individual bird served as the experimental unit. The proportional data were analyzed using the events/experiments syntax with a binomial distribution and both continuous and proportional data were analyzed using an R-side covariance structure. The pairwise least square mean comparisons were performed using the PDIFF option of SAS and considered different when *P* ≤ 0.05. Tendencies for differences among treatment means were declared when 0.0501 ≤ *P* ≤ 0.10.

## Results and Discussion

### Blood Plasma and Egg Yolk Vitamin D Concentrations

**Figure 2:**
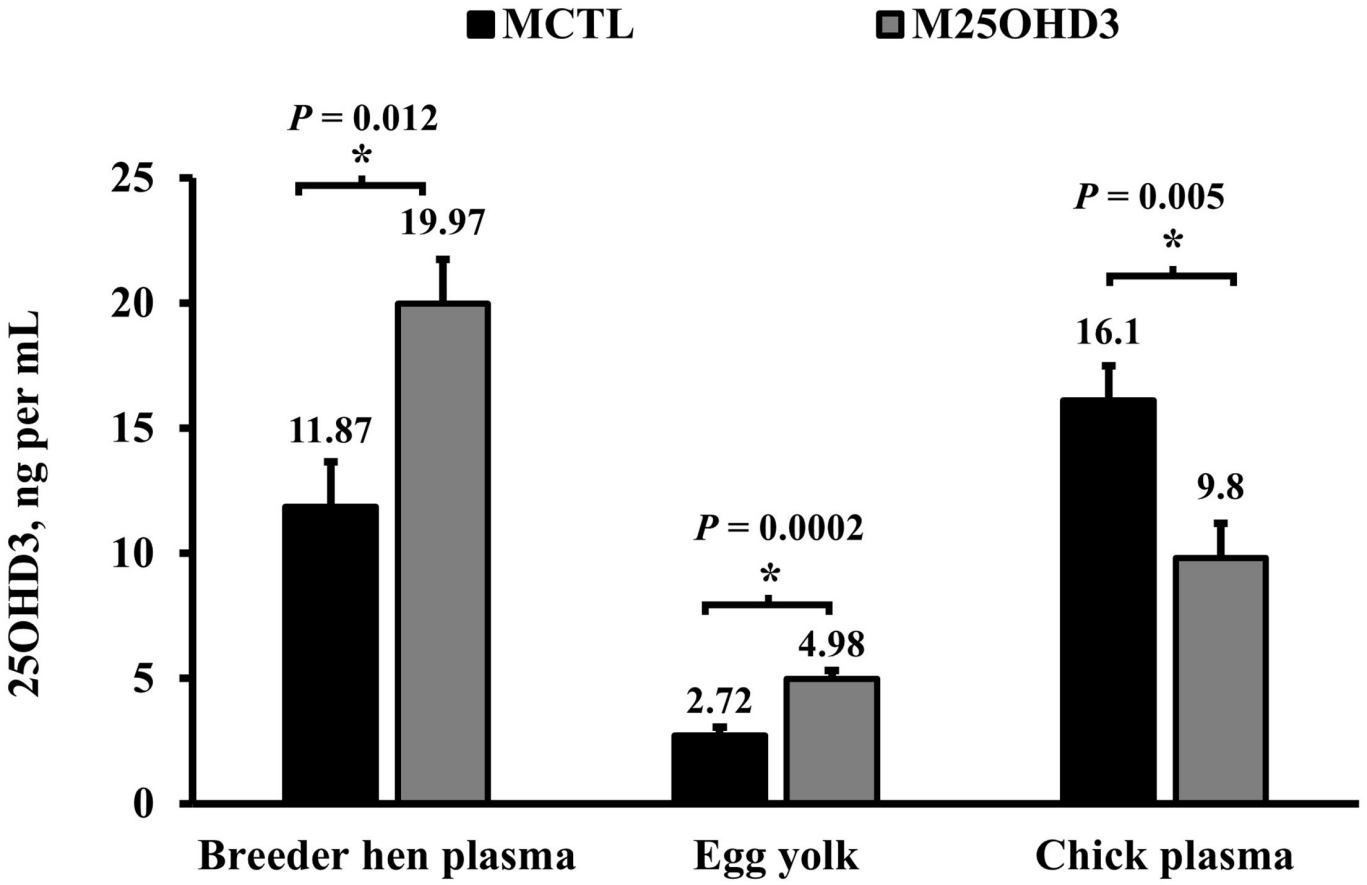
Effect of maternal 25-hydroxycholecalciferol dietary supplementation on breeder blood plasma, egg yolk, and hatched chick blood plasma 25-hydroxycholecalciferol concentration (Breeder plasma *n* = 10 total birds, 5 per treatment; egg yolk *n* = 20 total eggs, 10 per treatment; hatched chick plasma *n* = 10 total birds, 5 per treatment). Ross 708 broiler breeder pullets (*n* = 350, Aviagen Group, Huntsville, AL) were reared up to 21 weeks of age in a rearing facility and fed starter, developer, and pre-breeder diets without 25OHD3 metabolite in a skip a day restricted feeding program. After 25 weeks of age, broiler breeder hens and roosters were fed 1 of 2 corn and soybean-meal based maternal treatment diets (MDIET) 5,000 IU of D3 per kg of feed (MCTL; *n* = 5 pens), or 2,240 IU of D3 (Rovimix D3 500® DSM Nutritional Products Inc., Eggs and blood samples for analysis were collected on week 41 of breeder hen age. ^*^indicates means that differ at *P* ≤ 0.05.

In agreement with Mattila et al. (19), in our study (Figure 2), hen blood plasma 25OHD3 concentrations were higher in hens from the M25OHD3 treatment compared with hens from the MCTL group (19.97 vs. 11.87 ng per ml; *P* = 0.012). Maternal 25OHD3 supplementation (M25OHD3) improved egg yolk 25OHD3 concentrations in relation to eggs from breeder hens fed D3 only (4.98 vs. 2.72 ng per ml; *P* = 0.0002) these results are also comparable to those of Mattila et al. (19). Overall, our results support previous observations that indicate that 25OHD3 is effectively transferred from the diet to the hen's blood and egg yolks.

Even though eggs from hens fed the 25OHD3 diet had higher yolk 25OHD3 concentrations than eggs from hens fed D3 only, this was not reflected in hatched chick blood plasma 25OHD3 levels (Figure 2). Chicks from MCTL hens had higher blood plasma concentrations on the day of hatch than those from M25OHD3 hens (16.1 vs. 9.8 ng per ml; *P* = 0.005). The reasons why chick blood plasma 25OHD3 levels were higher in chicks from hens fed D3 only is unclear. Nevertheless, Saunders-Blades and Korver reported that hatched chick vitamin D status may be affected by breeder hen age (15).

In this study, the eggs were collected for incubation when breeder hens were 41 weeks old. Saunders-Blades and Korver reported a strong tendency during the mid-lay period (weeks 46 to 48) for chicks hatched from hens fed a diet supplemented with D3 only to have greater circulating 25OHD3 concentrations than chicks from hens fed a diet supplemented with 25OHD3 (38.70 vs. 27.33 ng per ml; *P* = 0.0564) (15). Considering the similarity in hen age of their study and ours (week 46–48 vs. week 41, respectively), higher concentration of circulating 25OHD3 in MCTL chicks compared with M25OHD3 chicks on the day of hatch can be attributed in part to the effect of hen age. In addition, since the yolk sac embodies an important nutrient reservoir for the chick and is intensely absorbed during the first 5 days post hatch (20), in addition to blood plasma 25OHD3 concentrations, yolk sac 25OHD3 concentrations could be a reliable indicator of chick vitamin D status at hatch.

**Figure 3:**
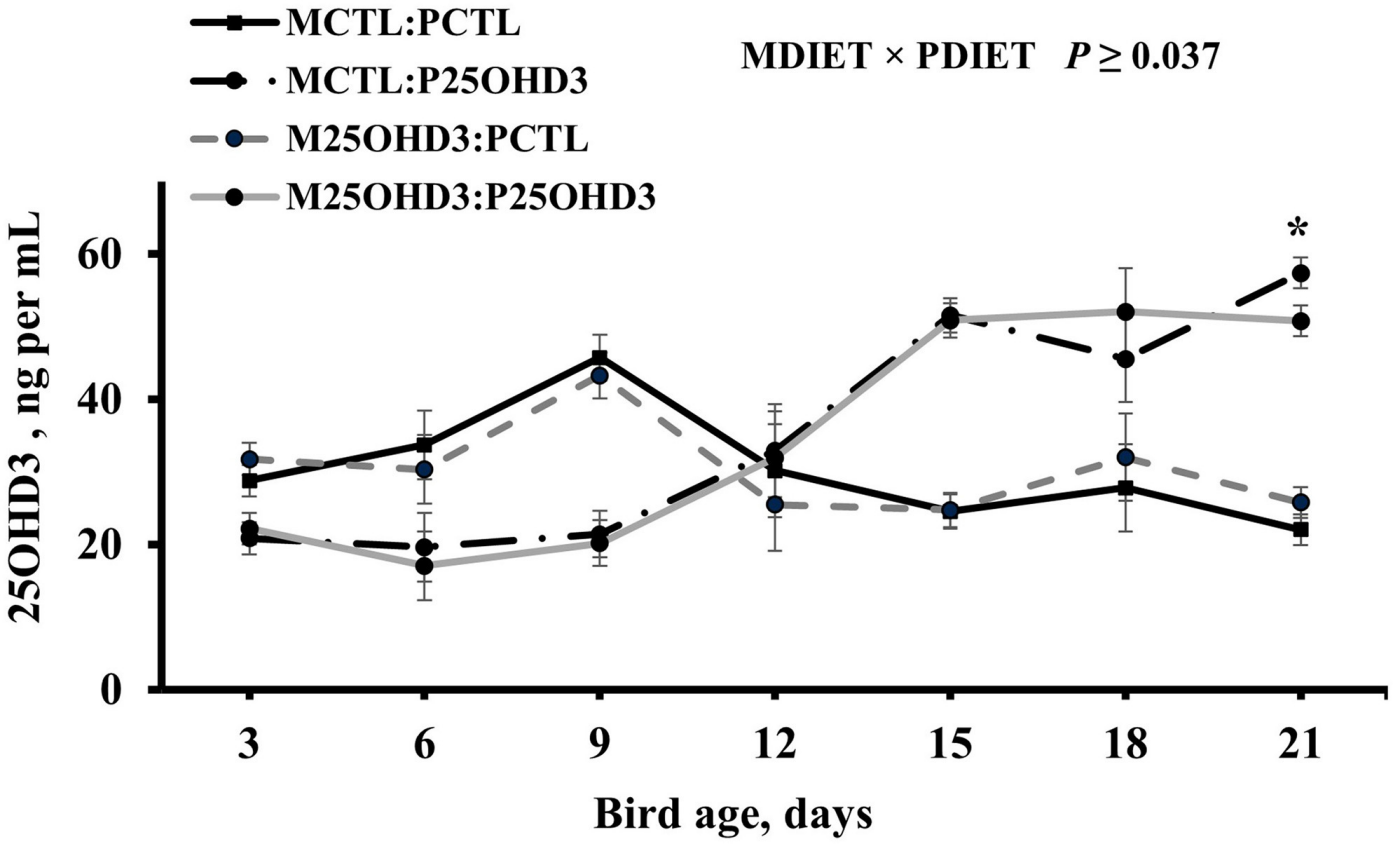
Effect of maternal and post-hatch dietary 25-hydroxycholecalciferol supplementation on broiler blood plasma 25-hydroxycholecalciferol concentrations. CTL diets = Diets containing 5,000 IU of D3 per kg of feed for either breeder hen (MCTL) or broiler (PCTL). 25OHD3 diets = diets containing 2,760 IU of 25OHD3 + 2,240 IU of D3 per kg of feed for breeder hen (M25OHD3) or broiler (P25OHD3). Treatment represents the combination of MDIET and PDIET, *n* = 20 birds total per sampling day, 5 birds per treatment per sampling day. Blood samples from chicks of both MDIET treatments were collected on day 0 and from the four MDIET × PDIET treatments on days 3, 6, 9, 12, 15, 18, and 21. *indicates means that differ at *P* ≤ 0.05.

On the day of hatch, chicks from the two maternal treatments (MCTL and M25OHD3) were randomly assigned to two post-hatch diets (PCTL and P25OHD3) that resulted in the four post-hatch treatments (MCTL:PCTL, MCTL:P25OHD3, M25OHD3:PCTL, and M25OHD3:P25OHD3). An interaction between MDIET and PDIET was observed at day 21 post-hatch (Figure 3) in which birds from the M25OHD3:P25OHD3 (50.8 ng per ml) and MCTL:P25OHD3 (57.4 ng per ml) treatments had higher blood plasma 25OHD3 concentrations than the MCTL:PCTL (22.1 ng per ml) and M25OHD3:PCTL (25.8 ng per ml) treatments (*P* = 0.037). No interactions between MDIET and PDIET were observed at any other sampling point.

**Figure 4:**
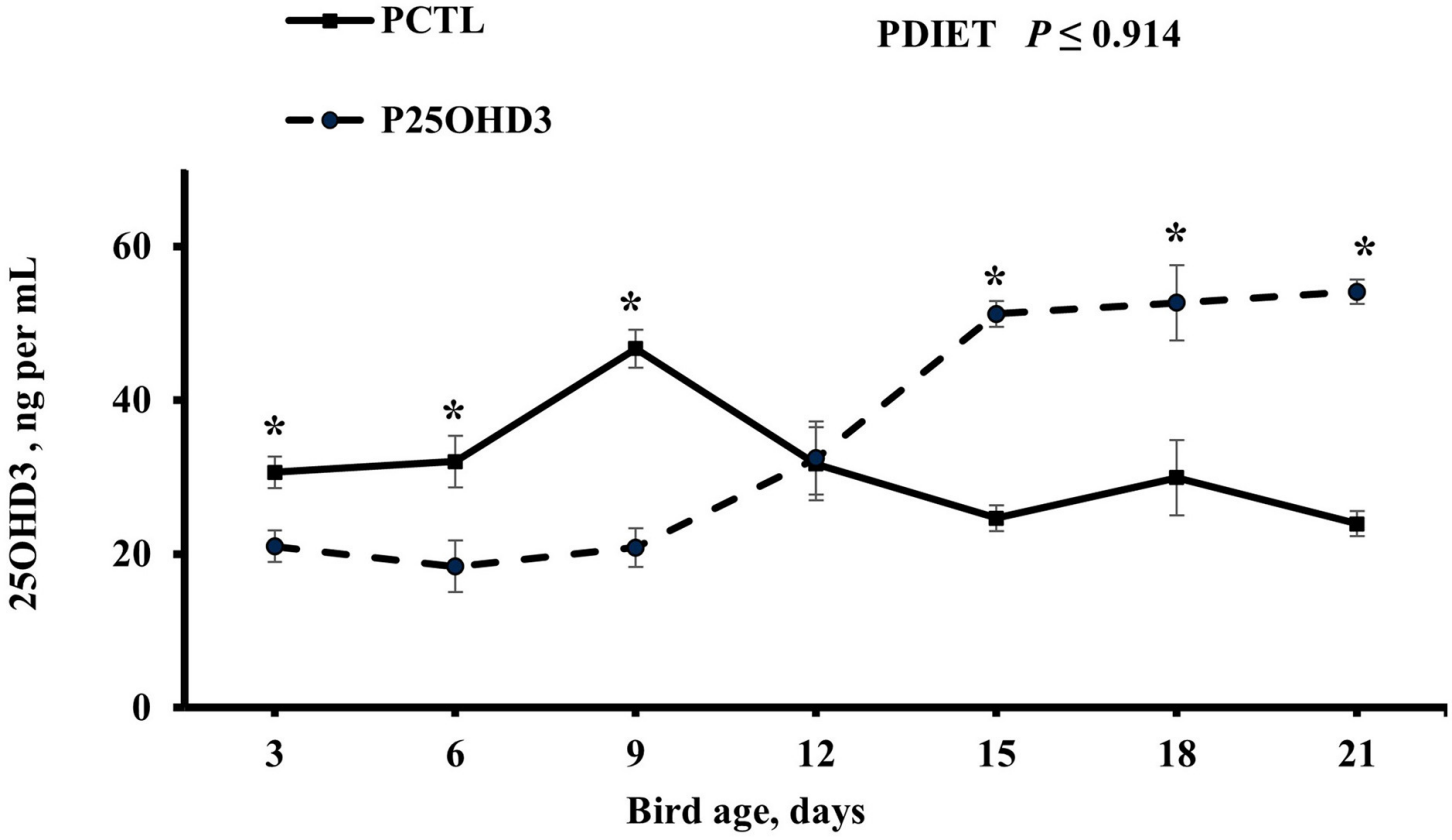
Effect of post-hatch dietary 25-hydroxycholecalciferol supplementation as a main effect on broiler blood plasma 25-hydroxycholecalciferol concentrations. CTL diets = Diets containing 5,000 IU of D3 per kg of feed (PCTL). 25OHD3 diets = diets containing 2,760 IU of 25OHD3 + 2,240 IU of D3 per kg of feed (P25OHD3). Treatment represents the combination of MDIET and PDIET, *n* = 20 birds total per sampling day, 10 birds per post-hatch treatment per sampling day. Blood samples from male broiler chicks were collected using cardiac puncture procedure at all ages. Blood was collected on days 3, 6, 9, 12, 15, 18, and 21. *indicates means that differ at *P* ≤ 0.05.

Maternal diet as a main effect did not impact circulating 250HD3 concentrations beyond day 0 (*P* ≥ 0.53). However, the post-hatch diet offered to chicks from day 0 to 21 had an interesting effect on chick circulating 25OHD3 levels (Figure 4). Blood plasma 25OHD3 concentrations were higher (*P* = 0.044) in PCTL than in P25OHD3 broilers from days 3 to 9, no difference was observed at day 12, and the effect observed during the first 9 days was reverted on d 15, 18, and 21 when P25OHD3 broilers had greater (*P* ≤ 0.005) blood plasma 25OHD3 concentration than PCTL broilers. Our findings contrast with the results of Hutton et al. (16) who observed higher concentrations of circulating vitamin D on broilers fed a diet containing a combination of 25OHD3 and D3 compared with birds fed a diet containing D3 as the only source of supplemental vitamin D as soon as day 7 post-hatch. In addition, the digestive capacity of avian gastrointestinal tract is not fully developed at hatch. Krogdahl and Sell observed that pancreatic amylolytic and proteolytic enzyme activities in poults increases rapidly during the first 28 days post-hatch while lipolytic enzyme activity increased slowly and did not reach maximum levels until 42–56 days after hatch (21). In chicks, biliary secretions are low during the immediate days post-hatch and may limit fat absorption (22). Therefore, fat soluble vitamin D may have not been absorbed efficiently during early days post-hatch and 25OHD3 plasma levels acquired during embryonic stage in addition to 25OHD3 stored in the yolk sack which is intensively absorbed during the first 5 days after hatch may represent an important fraction of vitamin D status of young chicks.

### Broiler Chicken Growth Performance

Contrary to hatched chick circulating vitamin D concentrations, maternal diet did not affect chick BW at the day of hatch (*P* = 0.764; Supplementary Table 3). The combination of maternal and post-hatch supplementation with 25OHD3 did not impact MCADBWG, MCFI, or MCADFI at any sampling point (days 3, 6, 9, 12, 15, 18, or 21; *P* ≥ 0.080). No interactions of MDIET and PDIET were observed for the variables BW or MCBWG at any sampling point except for day 15. At post-hatch day 15, significant interactions between MDIET and PDIET were observed (*P* = 0.023 and *P* = 0.022, respectively); however, for these variables, the *P*-value is significant as it is <0.05; however, there is no difference in the superscripts of treatments. In this case the *P*-value of the interaction is lower but very close to the established level of significance; however, *P*-values computed by multiple pairwise comparisons test are higher than the alpha significance level established (α = 0.05). Finally, birds from M25OHD3:P25OHD3 had a higher (worse) MCFCR at day 15 compared with birds from MCTL:P25OHD3 broilers (1.424 vs. 1.293; *P* = 0.004) as a result of a numerically lower MCBWG from days 0 to 15.

In literature, the effects of supplementing broiler diets with 25OHD3 on growth performance are conflicting. While some authors have observed improvements in body weight gain and feed conversion (7, 8, 23), others have not observed an impact of 25OHD3 inclusion in the diet on these growth performance parameters (10, 16, 24). In our study, since growth performance variables were calculated only for sampled birds (*n* = 12 per treatment per day, *n* = 48 total per day) it is likely that the number of birds was insufficient to detect small differences in performance among treatments for any sampling day.

### Duodenal Crypt Cell Proliferation

Intestinal crypts are the location of the small intestinal stem cells. These cells divide asymmetrically to produce a daughter cell and a new stem cell. Daughter cells proliferate as they migrate up the crypt. These proliferating undifferentiated cells are known as transit amplifying cells. The transit-amplifying cells differentiate into multiple cell types (enterocytes, goblet cells, Paneth cells, and enteroendocrine cells) as they exit the crypt and migrate up villi (25). To assess epithelial cell proliferation, two well oriented crypts per bird were selected and analyzed, crypt cell area was defined, and all cell nuclei (DAPI+) and proliferating cells (BrdU+) on epithelial lining were enumerated. These counts were then used to calculate the proportion of proliferating cells per crypt (PPC). In our study, due to their location in the epithelial lining of intestinal crypts and their state of proliferation, BrdU+ cells are most likely intestinal stem cells or transit amplifying cells.

**Figure 5:**
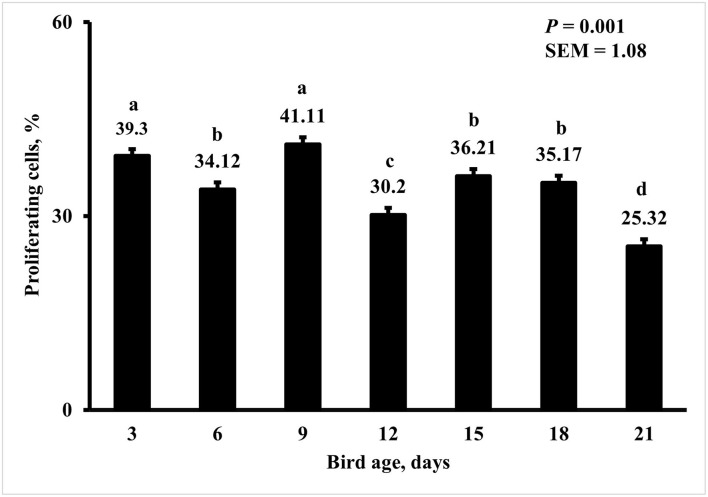
Effect of bird age on the proportion of proliferating cells (PPC) per duodenal crypt. On each sampling day, a total of 48 birds (*n* =12 per MDIET × PDIET treatment from 12 blocks) were selected for tissue collection. Samples were analyzed using a cryohistology and immunofluorescence procedure to label mitotically active cells (BrdU+) and total cell nuclei (DAPI+). To determine the proportion of proliferating cells per crypt, two well defined crypts were selected from each image (*n* = 2 per bird) the crypt area was defined by drawing a polygon around the epithelium. All cell nuclei and BrdU+ cells within the designated crypt were enumerated and the proportion of proliferating cells per crypt was then calculated by dividing the number of proliferating cells by the total number of cells in the crypt. ^a−d^Bars with different superscripts differ at *P* ≤ 0.05.

**Figure 6:**
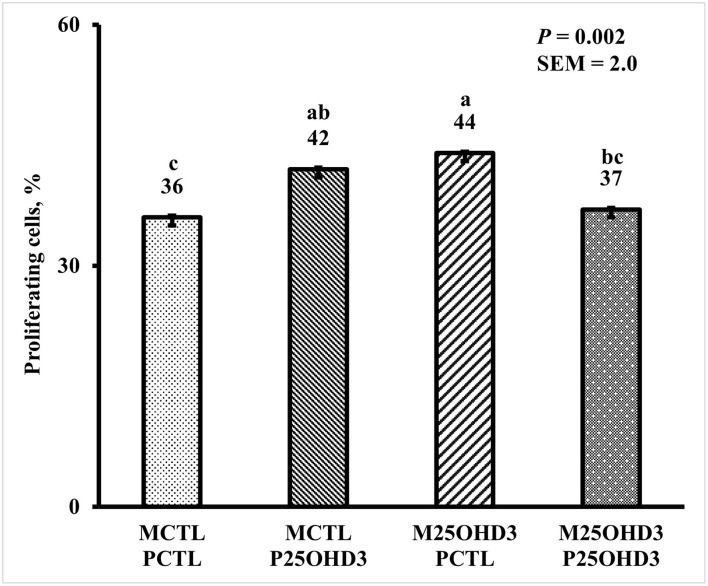
Effect of maternal and post-hatch dietary 25-hydroxycholecalciferol supplementation on day 3 broiler proportion of proliferating cells per duodenal crypt. Forty-eight birds were sampled on each sampling day (*n* = 12 of each MDIET × PDIET treatment). CTL diets = Diets containing 5,000 IU of D3 per kg of feed for either breeder hen (MCTL) or broiler (PCTL). 25OHD3 diets = diets containing 2,760 IU of 25OHD3 + 2,240 IU of D3 per kg of feed for breeder hen. (M25OHD3) or broiler (P25OHD3). Samples were analyzed using a cryohistology and immunofluorescence procedure to label mitotically active cells (BrdU+) and total cell nuclei (DAPI+). To determine proportion of proliferating cells per crypt, two well defined crypts were selected from each image (*n* = 2 per bird) the crypt area was defined by drawing a polygon around the epithelium. All cell nuclei and BrdU+ cells within the designated crypt were enumerated and proportion of proliferating cells per crypt was then calculated by dividing the number of proliferating cells by total number of cells in the crypt. ^a−c^Bars with different superscripts differ at *P* ≤ 0.05.

**Figure 7:**
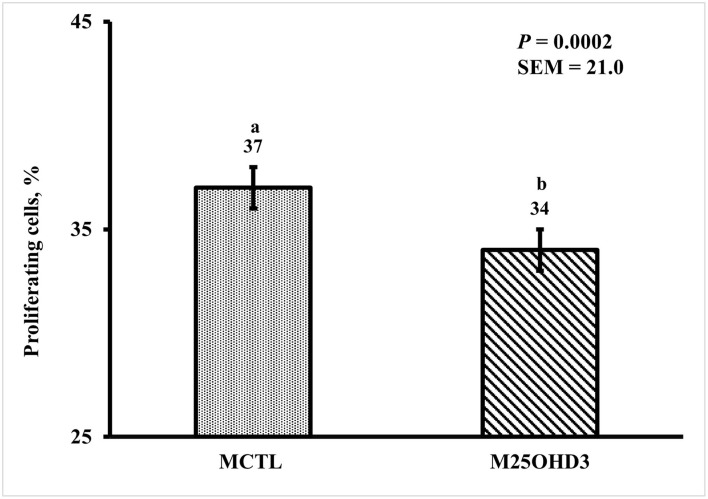
Effect of maternal dietary 25-hydroxycholecalciferol supplementation as a main effect on day 6 broiler proportion of proliferating cells per duodenal crypt. A total of 48 birds were sampled on each sampling day (*n* = 24 of each MDIET treatment). CTL diets = Diets containing 5,000 IU of D3 per kg of feed for breeder hen (MCTL). 25OHD3 diets = diets containing 2,760 IU of 25OHD3 + 2,240 IU of D3 per kg of feed for breeder hen (M25OHD3). Samples were analyzed using a cryohistology and immunofluorescence procedure to label mitotically active cells (BrdU+) and total cell nuclei (DAPI+). To determine the proportion of proliferating cells per crypt, two well defined crypts were selected from each image (*n* = 2 per bird) the crypt area was defined by drawing a polygon around the epithelium. All cell nuclei and BrdU+ cells within the designated crypt were enumerated and the proportion of proliferating cells per crypt was then calculated by dividing the number of proliferating cells by the total number of cells in the crypt. ^a−b^Bars with different superscripts differ at *P* ≤ 0.05.

Bird age impacted crypt PPC with the greatest PPC per duodenal crypt observed on days 3 and 9 (39 and 41%, respectively), and the lowest PPC per crypt was observed on day 21 (25%; *P* < 0.0001; Figure 5). A MDIET:PDIET interaction was observed on duodenal PPC at day 3 (Figure 6) where chicks from the M25OHD3:PCTL treatment had a higher PPC than those of the MCTL:PCTL treatment (44 vs. 36%; *P* = 0.002). No other interactions on duodenal PPC were observed beyond day 3; nevertheless, MDIET as a main effect impacted duodenal PPC at day 6 (Figure 7), being broilers from the MCTL treatment the ones with higher duodenal PPC compared with M25OHD3 birds (37 vs. 34%; *P* = 0.0002). Therefore, from our study, it appears that maternal supplementation of 25OHD3 may have a stronger effect on the modulation of duodenal crypt cell proliferation than post-hatch diet during the first week post-hatch.

Hence, the supplementation of broiler breeder diets with 25OHD3 may be beneficial since a greater proportion of proliferating cells is desired in early post-hatch stage to populate villi and ensure the functionality of epithelium for nutrient absorption, mucous, and hormone secretion. However, once the mucosa is fully developed, high epithelial cell proliferation requires more nutrients that are withdrawn from lean muscle accretion. Nevertheless, in the case of our study, it is unclear why maternal 25OHD3 increased proliferation especially since circulating 25OHD3 concentrations in M25OHD3 birds were lower at hatch than that of MCTL birds. However, it is also possible that 25OHD3 stimulates epithelial cells directly inducing the local production of 1,25OHD3 to act in an autocrine manner. It is also uncertain if this activity of 25OHD3 is mediated by genomic VDR pathway or non-genomic pathways.

Research suggests that vitamin D may play an important role in the development of morphology and functionality of chicken intestinal mucosa (26), especially through the regulation of epithelial cell proliferation. A previous study found that supplementing layer breeder diets with 25OHD3 resulted in taller villi in the three small intestinal segments of chicks on the day of hatch (14). Chou et al. observed that small intestines of broilers fed a diet containing 25OHD3 tended to be lighter at day 7 post-hatch than the small intestines of chicks fed D3 only (*P* < 0.1) (10). In addition, they found that feeding 25OHD3 resulted in longer villus height of duodenum at 21- and 28-day post-hatch and of jejunum of birds at 14 and 28 days of age (*P* < 0.05). Birds fed 25OHD3 had shorter duodenal crypt depth at day 14, 21, and 28 in jejunum (*P* < 0.05). As a result, birds from the 25OHD3 treatment had a higher villus to crypt ratio in duodenum and jejunum at days 14, 21, and 28 post-hatch (*P* < 0.05) (10).

How vitamin D modulates cell proliferation is still unclear; however, evidence suggests that it may be through effects on polyamine metabolism (26, 27). Polyamines (putrescine, spermidine, and spermine) have a significant role in cell proliferation and differentiation (28). An injection of 1,25OHD3, the most active vitamin D metabolite increased the conversion of ornithine and spermidine into putrescine in the duodenum of the D-deficient chicks (27). Furthermore, 1,25OHD3 enhances the activity of ornithine decarboxylase and spermidine N-acetyltransferase rate, limiting enzymes in the conversion of ornithine and spermidine into putrescine (27). Therefore, the modulation of duodenal epithelial cell proliferation by vitamin D, may be due to its effects on putrescine synthesis efficiency; nevertheless, further studies are required to fully elucidate this mechanism.

### Duodenal Macrophage Populations

Vitamin D in its biologically active form, 1,25OHD3, has a modulatory role in both innate and adaptive immunity (29). Activity of 1,25OHD3 is controlled by the VDR (30). The VDR is a transcription factor and performs its modulation of 1,25OHD3 genomic functions by forming heterodimers with the retinoid X receptor and regulating the expression of target genes by binding to the vitamin D response element in the promoter region (31). The expression of VDR in cells of both innate and adaptive immune systems such as monocytes, macrophages, dendritic cells, and T lymphocytes have been previously reported (32, 33). In addition, chicken macrophages express 1α-hydroxylase (11) allowing for the local synthesis of 1,25OHD3 from 25OHD3. However, in contrast with renal 1α-hydroxylase which is regulated by mediators of calcium and bone homeostasis such as PTH and 1,25OHD3 levels, macrophage 1α-hydroxylase has been reported to be regulated by immune signals like IFN-γ and IL-1 (34). The presence of VDR and 1α-hydroxylase D in macrophages suggests an important role of vitamin D in the modulation of innate immunity.

**Figure 8:**
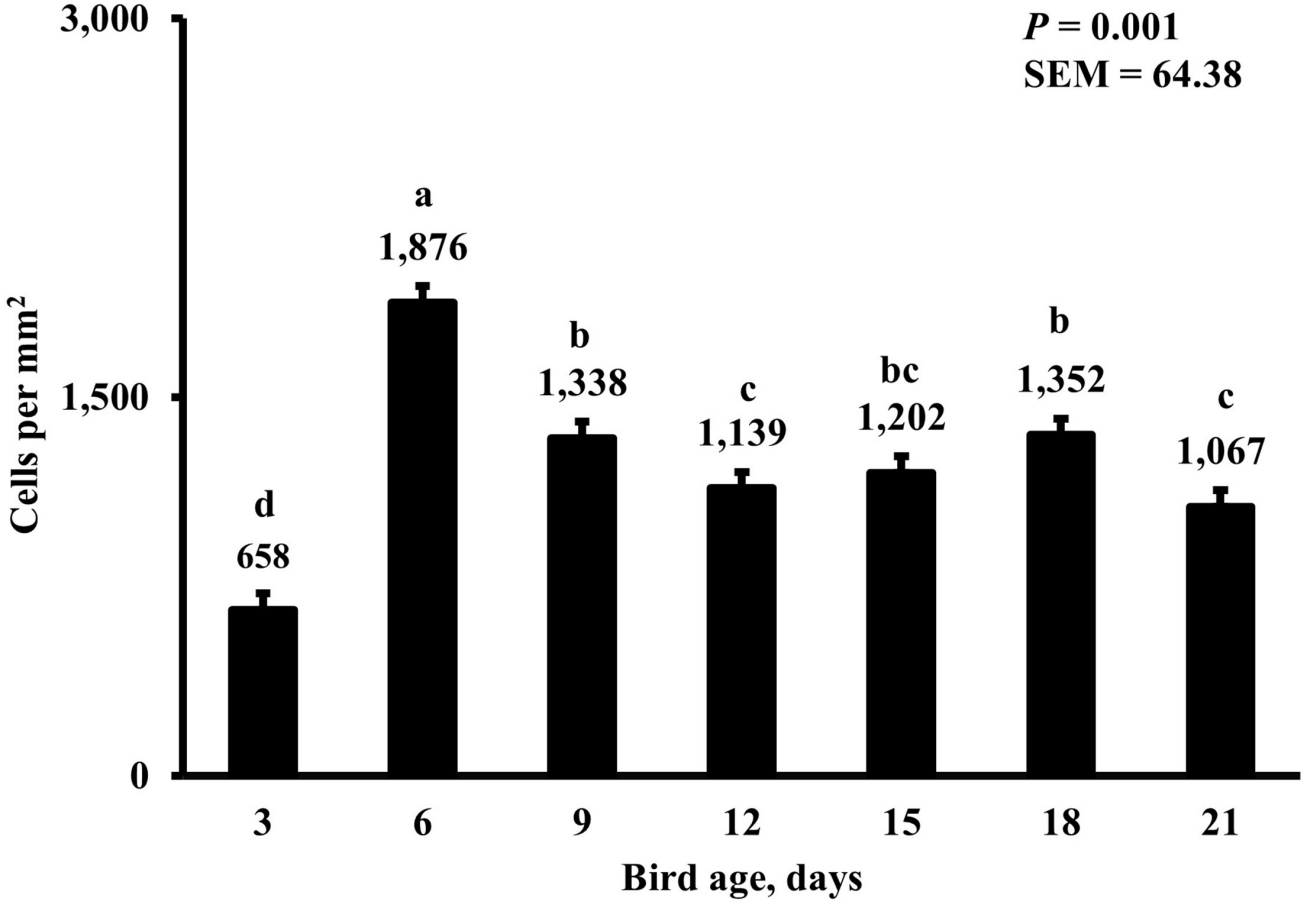
Effect of bird age on total duodenal macrophage density. This category represents the sum of all labeled macrophage populations in the duodenum. On each sampling day, a total of 48 birds (*n* =12 per MDIET × PDIET treatment from 12 blocks) were selected for tissue collection. ^a−d^Bars with different superscripts differ at *P* ≤ 0.05.

**Figure 9:**
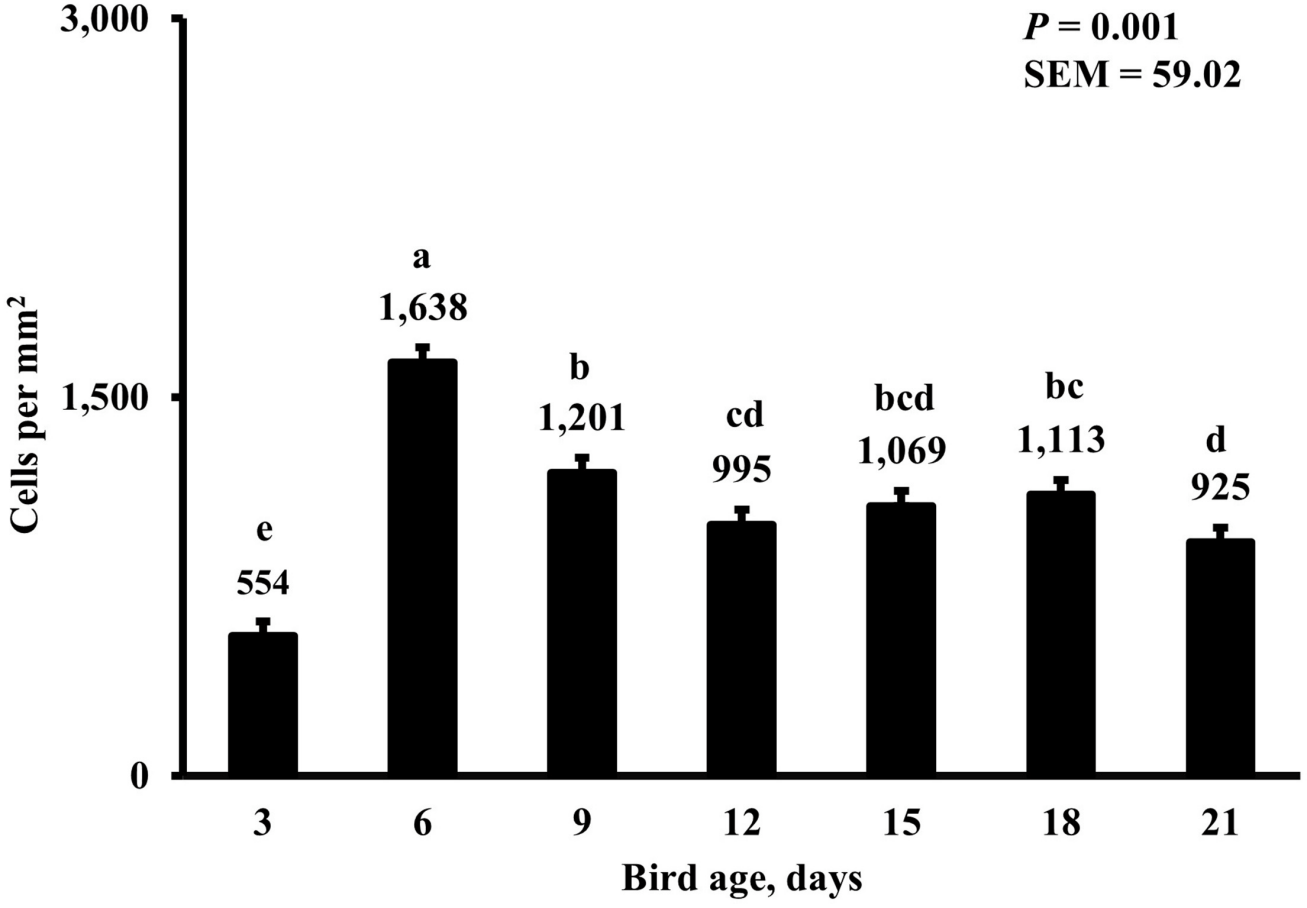
Effect of bird age on duodenal macrophage density. This category includes cells positive for KUL01 and DAPI only, it does not include proliferating or proinflammatory macrophages. On each sampling day, a total of 48 birds (*n* =12 per MDIET × PDIET treatment from 12 blocks) were selected for tissue collection. ^a−e^Bars with different superscripts differ at *P* ≤ 0.05.

**Figure 10:**
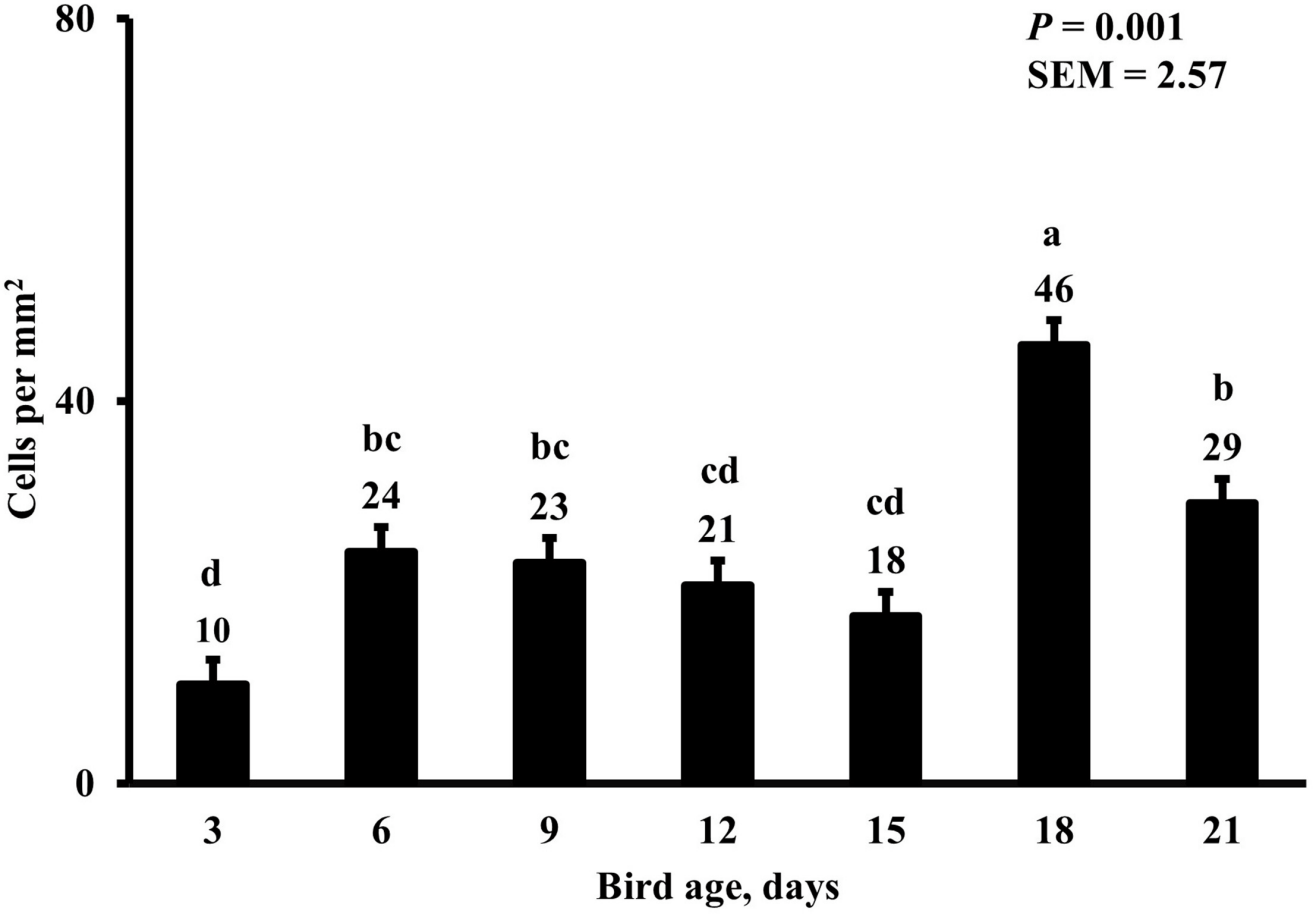
Effect of bird age on duodenal proliferating proinflammatory macrophage density. This category includes cells positive for KUL01, DAPI, CD80, and BrdU simultaneously. On each sampling day, a total of 48 birds (*n* =12 per MDIET × PDIET treatment from 12 blocks) were selected for tissue collection. ^a−d^Bars with different superscripts differ at *P* ≤ 0.05.

**Figure 11:**
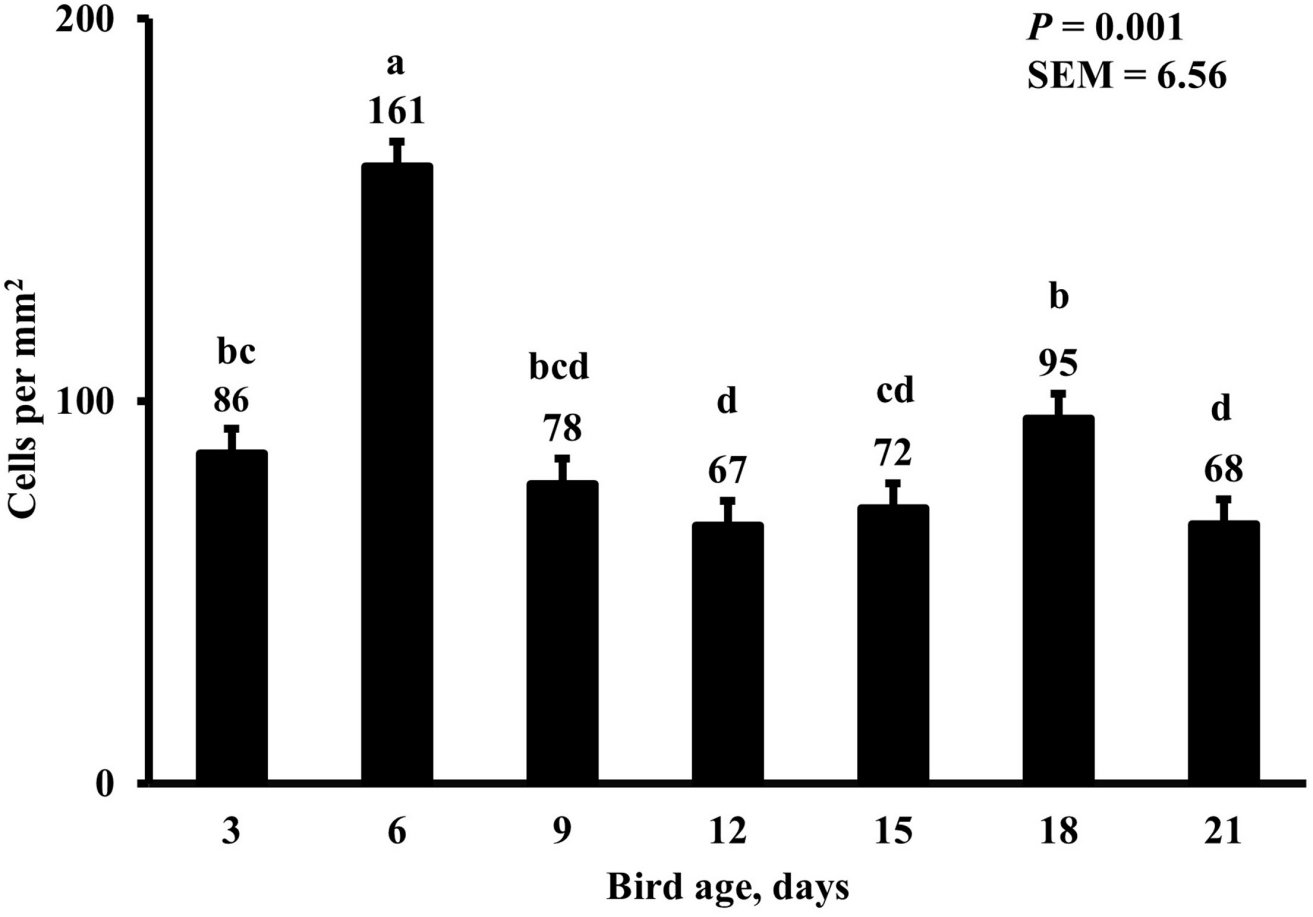
Effect of bird age on total duodenal proliferating macrophage density. This category represents the sum of all proliferating (BrdU+) macrophages. On each sampling day, a total of 48 birds (*n* =12 per MDIET × PDIET treatment from 12 blocks) were selected for tissue collection. ^a−d^Bars with different superscripts differ at *P* ≤ 0.05.

**Figure 12:**
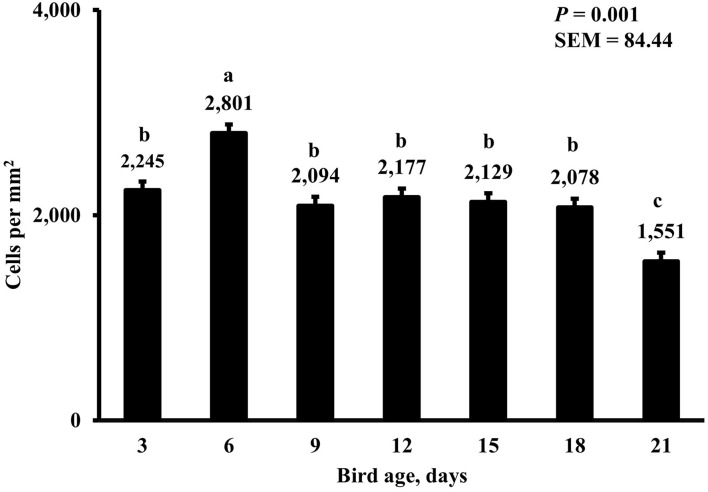
Effect of bird age on duodenal proliferating cell density. This category includes cells positive for BrdU and DAPI only, mainly epithelial cells. On each sampling day, a total of 48 birds (*n* =12 per MDIET × PDIET treatment from 12 blocks) were selected for tissue collection. ^a−c^Bars with different superscripts differ at *P* ≤ 0.05.

**Figure 13:**
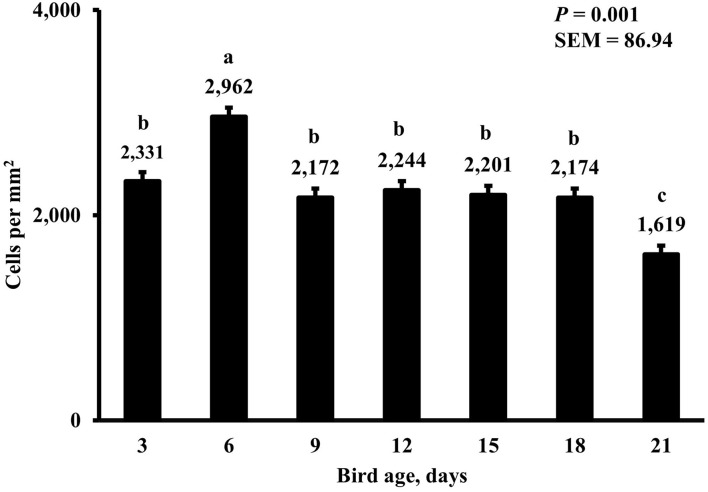
Effect of bird age on total duodenal proliferating cell density. This category represents all BrdU+ cells and includes macrophages and epithelial cells. On each sampling day, a total of 48 birds (*n* =12 per MDIET × PDIET treatment from 12 blocks) were selected for tissue collection. ^a−c^Bars with different superscripts differ at *P* ≤ 0.05.

**Figure 14:**
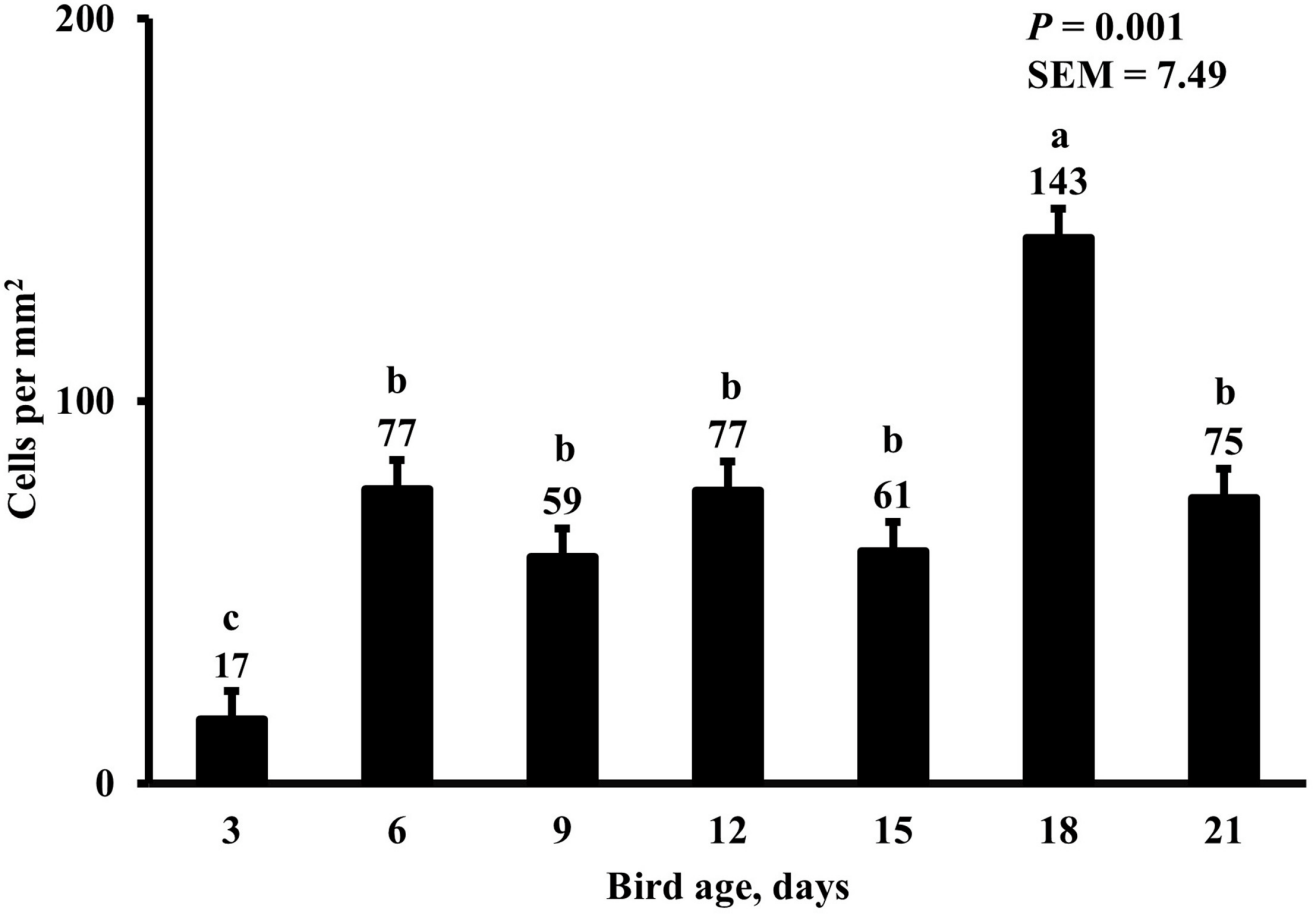
Effect of bird age on duodenal proinflammatory macrophage density. This category includes cells positive for KUL01, DAPI, and CD80 simultaneously. On each sampling day, a total of 48 birds (*n* =12 per MDIET × PDIET treatment from 12 blocks) were selected for tissue collection. ^a−c^Bars with different superscripts differ at *P* ≤ 0.05.

**Figure 15:**
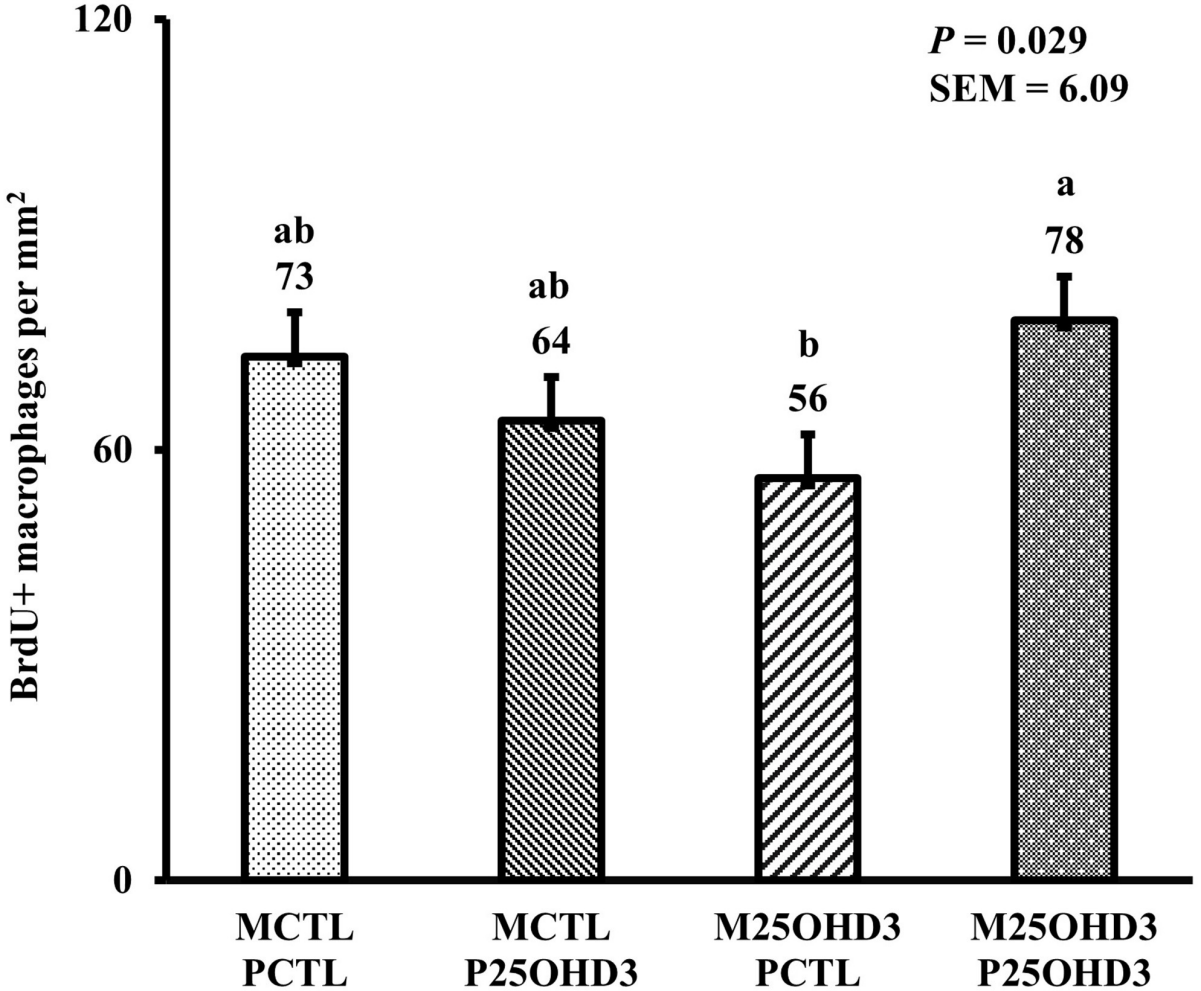
Effect of maternal and post-hatch dietary 25-hydroxycholecalciferol supplementation on day 21 broiler total proliferating duodenal macrophage density (BrdU+ cells per mm^2^). Birds (*n* = 48) were sampled on each sampling day (*n* = 12 of each MDIET × PDIET treatment). CTL diets = Diets containing 5,000 IU of D3 per kg of feed for either breeder hen (MCTL) or broiler (PCTL). 25OHD3 diets = diets containing 2,760 IU of 25OHD3 + 2,240 IU of D3 per kg of feed for breeder hen. (M25OHD3) or broiler (P25OHD3). ^a−b^Bars with different superscripts differ at *P* ≤ 0.05.

In the present study, the greatest density of total macrophages (Figure 8), macrophages (Figure 9), and proliferating, proinflammatory (Figure 10), total proliferating macrophages (Figure 11), proliferating cells (Figure 12), and total proliferating cells (Figure 13) were observed at day 6, while the density of proinflammatory macrophages was greatest at day 18 (Figure 14). It would be hard to attribute these effects to a particular reason since no intentional immune challenge was inflicted to the birds and other potential sources of intestinal inflammation were not measured. However, it is possible that the increase in total macrophages at day 6 could be explained by the exposure of intestinal mucosa to solid feed. It has been demonstrated that antinutrients present in feed ingredients such as non-starch polysaccharides (NSPs) can cause sustained inflammation in the small intestine (35). Interactions between MDIET and PDIET were observed in the present study. At day 21, an MDIET × PDIET interaction was observed where birds from the M25OHD3:P25OHD3 treatment had more proliferating macrophages than the M25OHD3:PCTL broilers (*P* = 0.029; Figure 15). Broiler chickens challenged with LPS were fed a diet supplemented with 69 μg/kg of 25OHD3 which resulted in lower expression of IL-1β mRNA in liver, and higher expression of 1α-hydroxylase in liver when compared with LPS challenged birds fed a diet supplemented with 3,000 IU per kg of vitamin D3 (12). Turkeys infected with *Eimeria maxima* coccidial oocysts and fed 110 μg/kg of 25OHD3 had 41% less fecal oocysts than infected turkeys fed 27 μg per kg of 25OHD3 at 5 days post-coccidial infection (13). Different from turkeys, no effect of 25OHD3 supplementation in fecal oocyst shedding was observed in layer hens (36). Broilers fed 25OHD3 in combination with CRINA®, a blend of essential oils, and vaccinated with viable sporulated oocysts from *E. maxima* and *E. tenella* had lower intestinal lesion scores induced by *E. tenella* compared with coccidia infected birds that were fed a diet containing no supplemental 25OHD3 (37). In addition, Morris et al. observed that supplementing diets with 100 μg per kg of 25OHD3 ameliorates reduction in body weight gain (BWG) induced by coccidial infection at 15 days post-infection in layers (36). Coccidia infected layers fed diets containing 6.25, 25, or 50 μg per kg of 25OHD3 had 15% BWG reductions compared with non-infected birds fed similar levels of 25OHD3, while birds fed 100 μg per kg of 25OHD3 had only 4% BWG reduction (36). It is apparent that 25OHD3 supplementation has more potent effect on immunity during immune challenges than during homeostatic conditions. Evidently, the inclusion of 25OHD3 in poultry diets increases immune competence, especially in the protection against coccidial and bacterial infection. The anti-inflammatory action of 25OHD3 may be the result of increased extrarenal activation of vitamin D as a result of an increased expression of 1α-hydroxylase and subsequent conversion of 25OHD3 to the active metabolite, 1,25OHD3, which can be a compensatory mechanism to reduce inflammation (12).

Overall, our findings indicate that combining maternal and post-hatch dietary supplementation with 25OHD3 affects duodenal crypt cell proliferation and intestinal macrophage populations with important effects on macrophage proliferation. Feeding supplemental 25OHD3 in poultry diets has previously been reported to aid during bacterial and parasitic infection, benefits of 25OHD3 supplementation may be accentuated during an immune challenge and may not be as evident in unchallenged conditions like those of our study. Future studies should be conducted to evaluate how combinations of maternal and post hatch 25OHD3 supplementation affects broiler intestinal innate immunity and intestinal development parameters. Finally, additional research should be performed to improve our understanding of the effects of breeder hen age and maternal vitamin D supplementation on 25OHD3 concentrations of broiler chicks at hatch and during the first week post-hatch.

## Data Availability Statement

The original contributions presented in the study are included in the article/Supplementary Material, further inquiries can be directed to the corresponding author.

## Ethics Statement

The animal study was reviewed and approved by the Auburn University Institutional Animal Care and Use Committee under Protocol No. 2017-3212.

## Author Contributions

SL, LA, GA-P, JF, KS, JW, CS, and JS conducted the experiments, analyzed the samples, and collected the data. SL, CS, and JS analyzed the data. SL wrote the original manuscript draft. JS and CS oversaw all experiments and revised the manuscript. All authors contributed to the article and approved the submitted version.

## Funding

The study was supported by the United States Department of Agriculture National Institute of Food and Agriculture (USDA-NIFA) through Hatch Act funds to the Alabama Agricultural Experiment Station.

## Conflict of Interest

The authors declare that the research was conducted in the absence of any commercial or financial relationships that could be construed as a potential conflict of interest.

## Publisher's Note

All claims expressed in this article are solely those of the authors and do not necessarily represent those of their affiliated organizations, or those of the publisher, the editors and the reviewers. Any product that may be evaluated in this article, or claim that may be made by its manufacturer, is not guaranteed or endorsed by the publisher.
