# Supplementary material for: Combined Maternal and Post-Hatch Dietary Supplementation of 25-Hydroxycholecalciferol Alters Early Post-Hatch Broiler Chicken Duodenal Macrophage and Crypt Cell Populations and Their Mitotic Activity
Source: Front Vet Sci. 2022 Apr 11;9:882566. doi: 10.3389/fvets.2022.882566 (PMC9036066; doi:10.3389/fvets.2022.882566)
Supplement: Supplementary file 1 [file Table_1.DOCX]

| **Supplementary Table 1. Composition of broiler breeder hen laying diets.** | | |
| --- | --- | --- |
| **Ingredients, %** | **Broiler breeder diet^1^** | |
|  | **MCTL** | **M25OHD3** |
| Ground corn | 60.39 | 60.39 |
| Soybean meal | 17.00 | 17.00 |
| Wheat middlings | 7.00 | 7.00 |
| Oats | 4.00 | 4.00 |
| Soybean oil^2^ | 1.30 | 1.30 |
| Limestone | 3.80 | 3.80 |
| Oyster shell (coarse) | 3.80 | 3.80 |
| Dicalcium phosphate | 1.70 | 1.70 |
| Salt | 0.30 | 0.30 |
| DL-Methionine | 0.19 | 0.19 |
| L-Threonine | 0.04 | 0.04 |
| Choline chloride 60% | 0.10 | 0.10 |
| Trace mineral premix^3^ | 0.10 | 0.10 |
| Vitamin premix^4^ | 0.03 | 0.03 |
| D3 premixed hand-add^5^ | 0.25 | - |
| 25OHD3 premixed hand-add^6^ | - | 0.25 |
| Total | 100.00 | 100.00 |
| **Calculated nutrients** |  |  |
| Vitamin D3, IU per kg of diet | 5,000.0 | 2,240.0 |
| 25-Hydroxycholecalciferol, IU per kg | - | 2,760.0 |
| Crude protein, % | 14.8 | 14.8 |
| Ca, % | 3.4 | 3.4 |
| P, % | 0.6 | 0.6 |
| Available P, % | 0.3 | 0.3 |
| ME, kcal per kg of diet | 2,735.0 | 2,735.0 |
| Digestible lysine, % | 0.6 | 0.6 |
| Digestible methionine, % | 0.4 | 0.4 |
| **Analyzed Nutrients** |  |  |
| Vitamin D3, IU per kg of diet | 4,413.0 | 1,760.0 |
| 25-Hydroxycholecalciferol, IU per kg | - | 1,732.0 |
| ^1^Fed to breeder hens and roosters starting wk 25. MCTL = maternal control diet; M25OHD3 = maternal 25-hydroxycholecalciferol diet (vitamin D contents below^5,6^). | | |
| ^2^Soybean oil included in mixer. | | |
| ^3^Supplied per kg of diet: Mn, 109 mg as Mn sulfate; Zn, 90 mg as Zn sulfate; Fe, 27 mg as ferrous sulfate; Cu 7 mg as basic Cu chloride; I, 1.3 mg as ethylenediamine hydroiodide; Se, 0.3 mg as sodium selenite. | | |
| ^4^Supplied per kg of diet: vitamin A, 11,000 IU; vitamin D3, 2,240 IU; vitamin E, 63 mg; vitamin K, 4.2 mg; thiamine, 3 mg; riboflavin, 12 mg; pyridoxin, 4.2 mg; cobalamin, 0.03 mg; niacin, 55 mg; pantothenic acid, 18.3 mg; folic acid, 2.8 mg; biotin, 0.46 mg. | | |
| ^5^Supplied per kg of diet: 2,760 IU of D3 in addition to 2,240 IU already provided^4^. | | |

| ^6^Supplied per kg of diet: 69 µg of 25-hydroxycholecalciferol equivalent to 2,760 IU. | | |
| --- | --- | --- |
| **Supplementary Table 2. Composition of broiler chicken post-hatch diets** | | |
| **Ingredients, %** | **Broiler post-hatch diet^1^** | |
|  | **PCTL** | **P25OHD3** |
| Ground corn | 50.96 | 50.96 |
| Soybean meal | 38.50 | 38.50 |
| Dried distiller grains | 3.00 | 3.00 |
| Soybean oil^2^ | 2.50 | 2.50 |
| Dicalcium phosphate | 1.80 | 1.80 |
| Limestone (fine) | 1.50 | 1.50 |
| Salt | 0.40 | 0.40 |
| DL-Methionine | 0.35 | 0.35 |
| L-Lysine | 0.15 | 0.15 |
| L-Threonine | 0.04 | 0.04 |
| Choline chloride 60 % | 0.10 | 0.10 |
| Trace mineral premix^3^ | 0.10 | 0.10 |
| No-D3 Vitamin premix^4^ | 0.05 | 0.05 |
| D3 premixed hand-add^5^ | 0.05 | - |
| 25OHD3 hand-add^6^ | - | 0.50 |
| Amprol® (amprolium, coccidiostat) | 0.05 | 0.05 |
| Total | 100.00 | 100.00 |
| **Calculated nutrients** |  |  |
| Vitamin D3, IU per kg of diet | 5,000.00 | 2,240.00 |
| 25-Hydroxycholecalciferol, IU per kg | - | 2,760.00 |
| Crude protein, % | 23.40 | 23.40 |
| Ca, % | 1.00 | 1.00 |
| Available P, % | 0.40 | 0.40 |
| ME, kcal per kg of diet | 2,940.00 | 2,940.00 |
| Digestible lysine, % | 1.20 | 1.20 |
| Digestible methionine, % | 0.60 | 0.60 |
| **Analyzed Nutrients** |  |  |
| Vitamin D3, IU per kg of diet | 4,390.00 | 2,144.00 |
| 25-Hydroxycholecalciferol, IU per kg | - | 1,978.00 |
| ^1^Starter (crumble): d 0 to 21. |  |  |
| ^2^Sprayed 1.5 % post-pellet. | | |
| ^3^Supplied per kg of diet: Mn, 109 mg as Mn sulfate; Zn, 90 mg as Zn sulfate; Fe, 27 mg as ferrous sulfate; Cu 7 mg as basic Cu chloride; I, 1.3 mg as ethylenediamine hydroiodide; Se, 0.3 mg as sodium selenite. | | |
| ^4^Supplied per kg of diet: vitamin A, 12,474 IU; vitamin E, 100 mg; vitamin K, 4 mg; thiamine, 3 mg; riboflavin, 8 mg; pyridoxin, 4 mg; cobalamin, 0.03 mg; niacin, 60 mg; pantothenic acid, 15 mg; folic acid, 2.5 mg; biotin, 0.4 mg. | | |
| ^5^Supplied per kg of diet: 5,000 IU of vitamin D3. | | |
| ^6^Supplied per kg of diet: 69 µg of 25-hydroxycholecalciferol equivalent to 2,760 IU. | | |
| \| **Supplementary Table 3. Effect of maternal and post-hatch dietary 25-hydroxycholecalciferol supplementation on broiler growth performance** \| \| \| \| \| \| \| \| --- \| --- \| --- \| --- \| --- \| --- \| --- \| \| **Variable^1^** \| **Dietary treatment combination^2^** \| \| \| \|  \|  \| \| \| **MCTL PCTL** \| **MCTL P25OHD3** \| **M25OHD3 PCTL** \| **M25OHD3 P25OHD3** \| **SEM** \| ***P-*Value** \| \| \| d 0 BW^3^, g \| 41.19^xy^ \| 41.40^x^ \| 41.56^x^ \| 40.87^y^ \| 0.27 \| 0.095 \| \| \|  \|  \|  \|  \|  \|  \|  \| \| \| *Sampling day*^4^ \|  \|  \|  \|  \|  \|  \| \| \| d 3 BW, g \| 83.42 \| 87.75 \| 89.17 \| 89.58 \| 2.99 \| 0.516 \| \| \| d 0-3 MCBWG, g \| 43.25 \| 46.33 \| 46.08 \| 48.33 \| 2.60 \| 0.873 \| \| \| d 0-3 MCADBWG, g \| 14.42 \| 15.44 \| 15.36 \| 16.11 \| 0.87 \| 0.874 \| \| \| d 0-3 MCFI, g \| 58.46^y^ \| 58.05^y^ \| 58.50^y^ \| 63.77^x^ \| 1.69 \| 0.080 \| \| \| d 0-3 MCADFI, g \| 19.47^y^ \| 19.35^y^ \| 19.50^y^ \| 21.26^x^ \| 0.51 \| 0.080 \| \| \| d 0-3 MCFCR \| 1.506 \| 1.348 \| 1.350 \| 1.397 \| 0.10 \| 0.322 \| \| \|  \|  \|  \|  \|  \|  \|  \| \| \| d 6 BW, g \| 149.42 \| 158.33 \| 147.33 \| 150.00 \| 4.57 \| 0.498 \| \| \| d 0-6 MCBWG, g \| 107.50 \| 115.83 \| 105.33 \| 109.92 \| 4.01 \| 0.642 \| \| \| d 0-6 MCADBWG, g \| 17.92 \| 19.31 \| 17.56 \| 18.32 \| 0.67 \| 0.642 \| \| \| d 0-6 MCFI, g \| 188.43 \| 175.58 \| 152.41 \| 171.11 \| 9.70 \| 0.100 \| \| \| d 0-6 MCADFI, g \| 31.50 \| 29.26 \| 25.40 \| 28.52 \| 1.62 \| 0.100 \| \| \| d 0-6 MCFCR \| 1.753^x^ \| 1.533^xy^ \| 1.459^y^ \| 1.582^xy^ \| 0.10 \| 0.096 \| \| \|  \|  \|  \|  \|  \|  \|  \| \| \| d 9 BW, g \| 244.17 \| 235.25 \| 241.17 \| 230.17 \| 7.89 \| 0.896 \| \| \| d 0-9 MCBWG, g \| 202.25 \| 194.33 \| 198.58 \| 189.92 \| 7.46 \| 0.960 \| \| \| d 0-9 MCADBWG, g \| 22.47 \| 21.59 \| 22.06 \| 21.10 \| 0.87 \| 0.874 \| \| \| d 0-9 MCFI, g \| 312.84 \| 292.98 \| 281.49 \| 284.24 \| 11.29 \| 0.322 \| \| \| d 0-9 MCADFI, g \| 34.76 \| 32.55 \| 31.28 \| 31.58 \| 1.25 \| 0.322 \| \| \| d 0-9 MCFCR \| 1.564 \| 1.524 \| 1.440 \| 1.513 \| 0.07 \| 0.434 \| \| \|  \|  \|  \|  \|  \|  \|  \| \| \| d 12 BW, g \| 362.08 \| 358.17 \| 376.92 \| 377.75 \| 8.98 \| 0.793 \| \| \| d 0-12 MCBWG, g \| 321.25 \| 316.75 \| 334.50 \| 336.33 \| 8.77 \| 0.720 \| \| \| d 0-12 MCADBWG, g \| 26.77 \| 26.40 \| 27.88 \| 28.03 \| 0.73 \| 0.719 \| \| \| d 0-12 MCFI, g \| 459.64 \| 439.20 \| 453.25 \| 473.34 \| 14.66 \| 0.161 \| \| \| d 0-12 MCADFI, g \| 38.30 \| 36.60 \| 37.77 \| 39.45 \| 1.17 \| 0.160 \| \| \| d 0-12 MCFCR \| 1.479 \| 1.394 \| 1.352 \| 1.414 \| 0.04 \| 0.106 \| \| \|  \|  \|  \|  \|  \|  \|  \| \| \| d 15 BW, g \| 511.92^a^ \| 542.67^a^ \| 541.75^a^ \| 507.17^a^ \| 13.88 \| 0.023 \| \| \| d 0-15 MCBWG, g \| 470.50^a^ \| 500.83^a^ \| 501.58^a^ \| 467.17^a^ \| 13.66 \| 0.022 \| \| \| d 0-15 MCADBWG, g \| 43.31 \| 43.17 \| 44.34 \| 44.17 \| 1.39 \| 0.991 \| \| \| d 0-15 MCFI, g \| 649.60 \| 647.58 \| 665.04 \| 662.48 \| 20.80 \| 0.990 \| \| \| d 0-15 MCADFI, g \| 43.31 \| 43.17 \| 44.34 \| 44.17 \| 1.39 \| 0.991 \| \| \| d 0-15 MCFCR \| 1.390^ab^ \| 1.293^c^ \| 1.324^bc^ \| 1.424^a^ \| 0.03 \| 0.004 \| \| \|  \|  \|  \|  \|  \|  \|  \| \| \| d 18 BW, g \| 744.42 \| 752.58 \| 731.25 \| 749.83 \| 18.93 \| 0.785 \| \| \| d 0-18 MCBWG, g \| 704.50 \| 712.75 \| 690.08 \| 708.33 \| 18.52 \| 0.789 \| \| \| d 0-18 MCADBWG, g \| 39.14 \| 39.60 \| 38.34 \| 39.35 \| 1.03 \| 0.789 \| \| \| d0-18 MCFI, g \| 940.66 \| 967.96 \| 904.37 \| 947.84 \| 26.18 \| 0.751 \| \| \| d0-18 MCADFI, g \| 52.26 \| 53.78 \| 50.24 \| 52.66 \| 1.45 \| 0.752 \| \| \| d0-18 MCFCR \| 1.342 \| 1.364 \| 1.310 \| 1.339 \| 0.02 \| 0.865 \| \| \|  \|  \|  \|  \|  \|  \|  \| \| \| d 21 BW, g \| 956.42 \| 963.08 \| 957.67 \| 985.17 \| 22.63 \| 0.648 \| \| \| d 0-21 MCBWG, g \| 914.58 \| 922.33 \| 915.00 \| 944.58 \| 22.54 \| 0.631 \| \| \| d 0-21 MCADBWG, g \| 43.55 \| 43.92 \| 43.57 \| 44.98 \| 1.07 \| 0.630 \| \| \| d 0-21 MCFI, g \| 1,523.38 \| 1,248.10 \| 1,227.91 \| 1,308.80 \| 36.23 \| 0.241 \| \| \| d 0-21 MCADFI, g \| 59.68 \| 59.43 \| 58.47 \| 62.32 \| 1.73 \| 0.241 \| \| \| d 0-21 MCFCR \| 1.3451 \| 1.3537 \| 1.3436 \| 1.3883 \| 0.03 \| 0.539 \| \| \| ^1^On an individual bird basis: BW = average body weight; MCBWG =Mortality corrected body weight gain; MCADBWG: Mortality corrected average daily body weight gain; MCFI = Mortality corrected feed intake; MCADFI = Mortality corrected average daily feed intake; MCFCR = Mortality corrected feed conversion ratio. \| \| \| \| \| \| \| \| ^2^CTL diets = Diets containing 5,000 IU of D3 per kg of feed for either breeder hen (MCTL) or broiler (PCTL). 25OHD3 diets = diets containing 2,760 IU of 25OHD3 + 2,240 IU of D3 per kg of feed for breeder hen (M25OHD3) or broiler (P25OHD3). \| \| \| \| \| \| \| \| ^3^n = 480 birds total (n = 120 per treatment). \| \| \| \| \| \| \| \| ^4^n = 48 birds sampled per day (*n* = 12 birds per treatment) on d 3, 6, 9, 12, 15, 18, and 21. \| \| \| \| \| \| \| \| ^ab^Means within a row with different letters are different at *P* < 0.05. \| \| \| \| \| \| \| \| ^xy^Means within a row with different are different at 0.0501≤ *P* ≤ 0.10. \| \| \| \| \| \| \| | | |
